# Progress of Surgery

**Published:** 1901-04-13

**Authors:** 


### PROGRESS OF SURGERY.

### OTOLOGY.

**The Production of Local Anæsthesia in the Ear.**—A satisfactory local anæsthetic, for use in acute inflammation of the middle ear, is much wanted, so that an incision can be made for the relief of pain without employing a general anæsthetic. Albert A. Gray,[1] after experiments with other vehicles—including rectified spirit and volatile oils, which dissolve cocaine and penetrate the tissues quickly—finally tried a mixture of aniline oil and dilute alcohol in equal parts. Cocaine is dissolved in this to a strength of a little less than 5 per cent. He found this solution acted admirably, as it penetrated the tissues rapidly without destroying them, and was miscible with water to a considerable extent. The writer remarks that to penetrate the outer layers of the tympanic membrane dehydrating agents are the most suitable. Alcohol and aniline oil are both substances of this description. The above solution is suitable for operations on the tympanic membrane, on granulations, or for the removal of ossicles. If a dense and thick membrane has to be operated upon the penetrating power of the solution may be increased by using absolute alcohol in place of rectified spirit and increasing the proportion of aniline oil. The external meatus should be filled with the solution. Serious effects of cocaine poisoning need not be feared. The same solution is better than an aqueous one of cocaine for throat work. The mixture of oil and spirit without cocaine is beneficial in suppurative affections of the middle ear.

**Useful Hearing obtained in a Deaf Mute, aged 19 Years.**—J. Lockhart Gibson,[2] of Brisbane, Q., has published notes of this case which show the importance of not concluding too readily that a deaf mute, with some hints that hearing has not been absolutely destroyed, should be left severely alone, and of removing lymphoid hypertrophies in the naso-pharynx. The author believes that comparatively small hypertrophies in the fossæ of Rosenmüller are apt to do more harm to the hearing than larger ones in the general naso-pharyngeal space. The history was that the patient could hear as a baby. She had begun to talk a little when, at the age of 18 months, she had measles. After that she did not talk until taught lip reading. She had been six years in a deaf and dumb institution. Her teaching was helped a little by using an ear trumpet, but for all practical purposes she was deaf and dumb. A considerable amount of hypertrophied tissue was removed from the naso-pharynx, especially from the fossæ of Rosenmüller, under cocaine. Within a month there was a great improvement in the hearing. The ears were regularly politzerised after the operation.

**Traumatic Rupture of the Membrana Tympani.**—Richards [2] records a case of this injury in a fireman who was struck on the head at short range by the stream from a hose. He was stunned, and a few hours later the ear began to discharge. He was seen three days later and complained then chiefly of tinnitus, and was treated with iodoform insufflations. Similar cases have been reported of rupture of the membrane from waves striking the head in bathing, and from concussion in heavy gun firing. Several cases occurred amongst the sailors working the turret guns during the bombardment of Santiago. The symptom most complained of in all cases was tinnitus. The loss of hearing was not of extreme degree, and only of temporary duration.

**Acute Otitis Media.**—Charles M. Cox [3] contributes an article on this subject. He remarks on the custom of describing two kinds of acute inflammation of the middle ear, viz., the catarrhal and the suppurative. In the former resolution frequently takes place without rupture of the membrana tympani. After rupture of the membrane it is difficult to say whether the inflammation was suppurative from the beginning or not. Acute suppuration is met with at all ages, but by far the most frequently in childhood. Of predisposing causes the chief and foremost is the presence of adenoids in the naso-pharynx. Even small lymphoid masses, which cause no obstruction to nasal breathing, if in a state of chronic inflammation, predispose to acute otitis. When tuberculous infection of the middle ear has taken place there is the peculiarity that the suppurating process lacks the acuteness by which it is usually characterised. There is then little, if any, pain; the tympanic cavity is pale and œdematous in appearance, and the inflammation tends to assume a chronic form from the beginning. Diabetes is sometimes a cause. The view is taken that bathing excites acute otitis by causing water to be inadvertently drawn into the naso-pharynx and then forced up the Eustachian tube, rather than by causing it to enter the external meatus. In the early stages the pain of the affection is markedly increased by pressure upon the tongue. Otitis should always be thought of as a possible cause of convulsions in children. The peculiar cry of a child with this disease is regarded as almost pathognomonic. It is a piercing continuous cry which is kept up without a moment's cessation until the child is exhausted, or the pain mitigated for the time. The drum usually perforates in the antero-inferior portion. Extension to the labyrinth is rather unusual. It may be suspected if there be marked dizziness, nausea, and absolute deafness for all or some, usually the higher, notes. If a perforation cannot be seen, but a little pulsating light reflex deep down in the canal is visible, this is practically a positive proof of perforation. As a final test the gentle inflation with the Politzer bag, or by Valsalva's method will generally elicit the so-called "perforation whistle," and then minute air bubbles will be seen in the canal. It may be impossible to force air through the opening if it is in the membrana flaccidi, or Shrapnell's membrane. Stephen H. Lutz, in the same publication, discusses the treatment of this affection. In the beginning the object is to prevent the formation of pus. For the pain, the best thing to use is hot water, either poured, or gently syringed, into the canal. Leeches can be used in front of the tragus, and over the mastoid close to the auricle, to relieve congestion of the membrane and canal wall. In paracentesis the knife is plunged into the most bulging portion of the tympanic membrane, carried up well into the attic, and then swept downwards and backwards in a crescent along the bony canal wall, using a fair amount of pressure. Nitrous oxide anæsthesia may be used, cocaine being of very little value. It is often difficult to keep the incision open as it heals so quickly. Frequent douching must be maintained. Tonsils and adenoids are best removed when the discharge has ceased, or at any rate quieted down. The writer does not like the use of powders. When thin discharge persists he uses hydrastis, zinc sulphate, protargol, or nitrate of silver solutions. B. Heine [4] has written a paper on the special danger of acute purulent ear suppuration in elderly people. He gives particulars of several cases with fatal results. The symptoms are not well marked, and the signs of mastoid disease very indefinite. Sclerosis of the bone exists, and suppuration progresses towards the top of the petrous portion of the temporal bone, and may be missed there at the operation. More frequent operation is recommended in elderly people who have pain over the mastoid, depression of the posterior superior wall of the auditory meatus, or severe pain on one side of the head. Alexander Francis [5] (Brisbane) contributes a note upon a case of emphysematous otitis due to the bacillus aërogenes capsulatus. He believes no similar case has been recorded, and compares it to emphysematous vaginitis, the pathology of which was elucidated by the discovery of the bacillus in 1892. The patient, a woman, was suddenly seized with acute pain in the left ear, which in a few hours became agonising. The membrane, which was bulging, was punctured. Clear fluid bubbled out, and the pain was relieved at once. About fifteen hours afterwards pain began in the right ear, and a large bulla formed in the posterior wall of the external auditory meatus, the drum remaining normal. This was punctured, a quantity of clear fluid fizzed out, making a noise which was heard some yards away, and the canal filled with bubbles. The pain was relieved immediately, but some hours later it returned again, and was so severe that it almost drove the patient out of her mind. The trouble this time was behind the drum, in the middle ear. The fact that the bacillus assumed to be the cause of the disease was anærobic, suggested to Francis the use of peroxide of hydrogen. This yielded very satisfactory results, as after its employment there was no further trouble. Though there was considerable destruction of tissue, the drum membranes healed perfectly and the hearing became almost normal. Cover-glass specimens of the fluid showed the presence of the bacillus in considerable numbers.

**Otitis Interna.**—Körner [4] has contributed a paper on the surgical treatment of suppuration in the labyrinth. Middle ear suppuration not infrequently spreads into the internal ear. Owing to the connections the suppuration then readily spreads to the posterior cranial fossa and leads to meningitis or cerebellar abscess. The suppuration in the labyrinth does not always spread to all its parts; in the cochlea the disease may be shut off by granulations from the vestibule, and in the vestibule or semicircular canals it may similarly be shut off from the cochlea. The commonest seat of attack is the horizontal semicircular canal and the vestibule. The symptoms then are sudden attacks of vomiting and giddiness with nystagmus, and the patient falls to the side of disease. When the different middle ear spaces are laid open fistulæ may be seen leading to the semicircular canal and vestibule, and these can be followed; or the vestibule may be opened, even if there are no fistulæ, if the symptoms point to disease there. The author describes two cases in which he successfully operated on the vestibule for suppuration. Charles Ballance [6] has also reported a case of this kind which he operated upon successfully, removing a serious menace to the life of the patient and restoring useful hearing power to an absolutely deaf ear. The patient, a woman aged 54, had suffered from otorrhœa since childhood. In 1898 Ballance had performed the radical mastoid operation. But vertigo, otorrhœa, and absolute deafness had persisted. In 1900, therefore, the operation in question was performed. A sinus was found leading into the petrous portion of the bone and this was enlarged, parts of the semicircular canals removed, the vestibule opened, and the cavity swabbed out with absolute phenol. Ten days later the cavity was skin-grafted. When the gauze packing was removed, five days after the operation, the patient said she could hear well, and two and a half months later the wound was soundly healed, the hearing good, and the patient free from giddiness and tinnitus. Jobson Horne [6] has described two cases in which labyrinthine suppuration spread down the minute canal leading to the posterior fossa (the aqueductus vestibuli), and a small abscess had formed at its opening between the layers of the dura mater covering the posterior surface of the petrous bone, in the site of the saccus endolymphaticus. Thus the aqueductus vestibuli is capable of transmitting septic infection. In both cases a minute rupture of the abscess had infected the meninges of the posterior fossa and the lateral sinus. In operations for intracranial complications of ear disease, Horne recommends that this region of the dura mater should be carefully examined for the presence of an abscess.

**Facial Paralysis in Acute Otitis Media.**—William R. Murray [7] (Minneapolis) states that out of 258 cases of acute otitis which were treated during one year in the Illinois Eye and Ear Infirmary, there were two cases complicated with facial paralysis. The first case was that of a man of 34, who received a kick on the left ear, followed by severe pain in the ear persisting until the drum ruptured and purulent discharge escaped. Facial paralysis was also present. At the end of six weeks all signs of the paralysis were gone. It is not stated whether the facial paralysis immediately followed the kick. The second patient was a schoolboy who had had severe earache for three weeks, accompanied by a left facial paralysis. The membrane was found perforated, and muco pus exuded from the tympanum. The ear healed and the paralysis disappeared under treatment.

[1] Vide Abst. Jour. Laryng. of R. and O., Nov. 1900. [2] Vide Abst. Jour. L.R. and O., Jan. 1901. [3] Brooklyn Med. Jour., Nov. 1900. [4] Vide Abst. Jour. L.R. and O., Dec. 1900. [5] Australasian Med. Gazette, Oct. 1900. [6] Vide Abst. Quarterly Jour. Med. Sciences, Nov. 1900. [7] Vide Abst. Glasgow Med. Jour., Oct. 1900.

